# Osteopontin facilitates West Nile virus neuroinvasion via neutrophil “Trojan horse” transport

**DOI:** 10.1038/s41598-017-04839-7

**Published:** 2017-07-05

**Authors:** Amber M. Paul, Dhiraj Acharya, Laurel Duty, E. Ashley Thompson, Linda Le, Dobrivoje S. Stokic, A. Arturo Leis, Fengwei Bai

**Affiliations:** 1 Department of Biological Sciences, The University of Southern Mississippi, Hattiesburg, MS 39406 USA; 2 100002401 Center for Neuroscience and Neurological Recovery, Methodist Rehabilitation Center, Jackson, MS 39216 USA; 3 Department of Neurology, Mayo Clinic, Scottsdale, AZ 85259 USA

## Abstract

West Nile virus (WNV) can cause severe human neurological diseases including encephalitis and meningitis. The mechanisms by which WNV enters the central nervous system (CNS) and host-factors that are involved in WNV neuroinvasion are not completely understood. The proinflammatory chemokine osteopontin (OPN) is induced in multiple neuroinflammatory diseases and is responsible for leukocyte recruitment to sites of its expression. In this study, we found that WNV infection induced OPN expression in both human and mouse cells. Interestingly, WNV-infected OPN deficient (*Opn* ^−/−^) mice exhibited a higher survival rate (70%) than wild type (WT) control mice (30%), suggesting OPN plays a deleterious role in WNV infection. Despite comparable levels of viral load in circulating blood cells and peripheral organs in the two groups, WNV-infected polymorphonuclear neutrophil (PMN) infiltration and viral burden in brain of *Opn* ^−/−^ mice were significantly lower than in WT mice. Importantly, intracerebral administration of recombinant OPN into the brains of *Opn* ^−/−^ mice resulted in increased WNV-infected PMN infiltration and viral burden in the brain, which was coupled to increased mortality. The overall results suggest that OPN facilitates WNV neuroinvasion by recruiting WNV-infected PMNs into the brain.

## Introduction

West Nile virus (WNV) is a single-stranded RNA virus belonging to the flaviviridae family that is primarily transmitted to humans by infected mosquitos^1, 2^. Although the majority of WNV-infected individuals remain asymptomatic, some develop symptoms ranging from mild fever and maculopapular rash to severe neuroinvasive diseases, including flaccid paralysis, encephalitis, meningitis, and possible death. The pathogenesis of neuroinvasive WNV is still not well understood and there is no vaccine or specific antiviral therapeutic available for human use.

Osteopontin (OPN) is a multifunctional protein also known as early T-lymphocyte activation-1 (ETA-1) and secreted phosphoprotein-1 (SPP-1)^3, 4^. OPN is produced by a variety of immune cells, including mast cells, dendritic cells, macrophages, neutrophils, B lymphocytes and T lymphocytes^5, 6^. There are two primary protein isoforms of OPN, intracellular (iOPN) and secreted (sOPN) OPN, each having a distinct function in the context of immunity. For instance, iOPN is involved in amplification of the type I interferon (IFN) response following Toll-like receptor (TLR) engagement^7, 8^, whereas sOPN can recruit leukocytes to sites of its expression^9, 10^. OPN has a wide range of biological functions, such as biomineralization and bone remodeling^3^, but it is also involved in the pathogenesis of many neuroinflammatory diseases, including multiple sclerosis^11^, Parkinson’s disease^12^, Alzheimer’s disease^13^ and in cancer cell metastases and brain tumor formation^14^. OPN expression is also induced by various viral infections, including human immunodeficiency virus^15^, hepatitis C virus^16^ and Zika virus infection in human neural progenitor cells^17^. However, the role of OPN during WNV pathogenesis is not known.

Polymorphonuclear neutrophils (PMN) constitute approximately 50–70% of the total human leukocyte population and are the first immune cells recruited to sites of infection^18, 19^. PMNs are crucial players in controlling bacterial and fungal infections through phagocytosis, degranulation and neutrophil extracellular traps (NETs)^18^. We have previously shown that PMNs were permissive to WNV infection and harbored 75% of WNV in the total leukocyte population of mice^20^, thus it is possible that WNV-infected PMNs play an important role in transporting the virus into the central nervous system (CNS), contributing to neuroinvasive disease. In line with this, human WNV cases with neurological disease have increased neutrophils in their cerebral spinal fluid (CSF), indicating a possible role for PMNs in the development of the neurological symptoms of WNV infection^21, 22^. In this study, we show that sOPN promotes WNV-infected PMN recruitment into the CNS, enhancing disease severity.

## Results

### WNV infection induces OPN expression in both human and mouse cells

**Figure 1:**
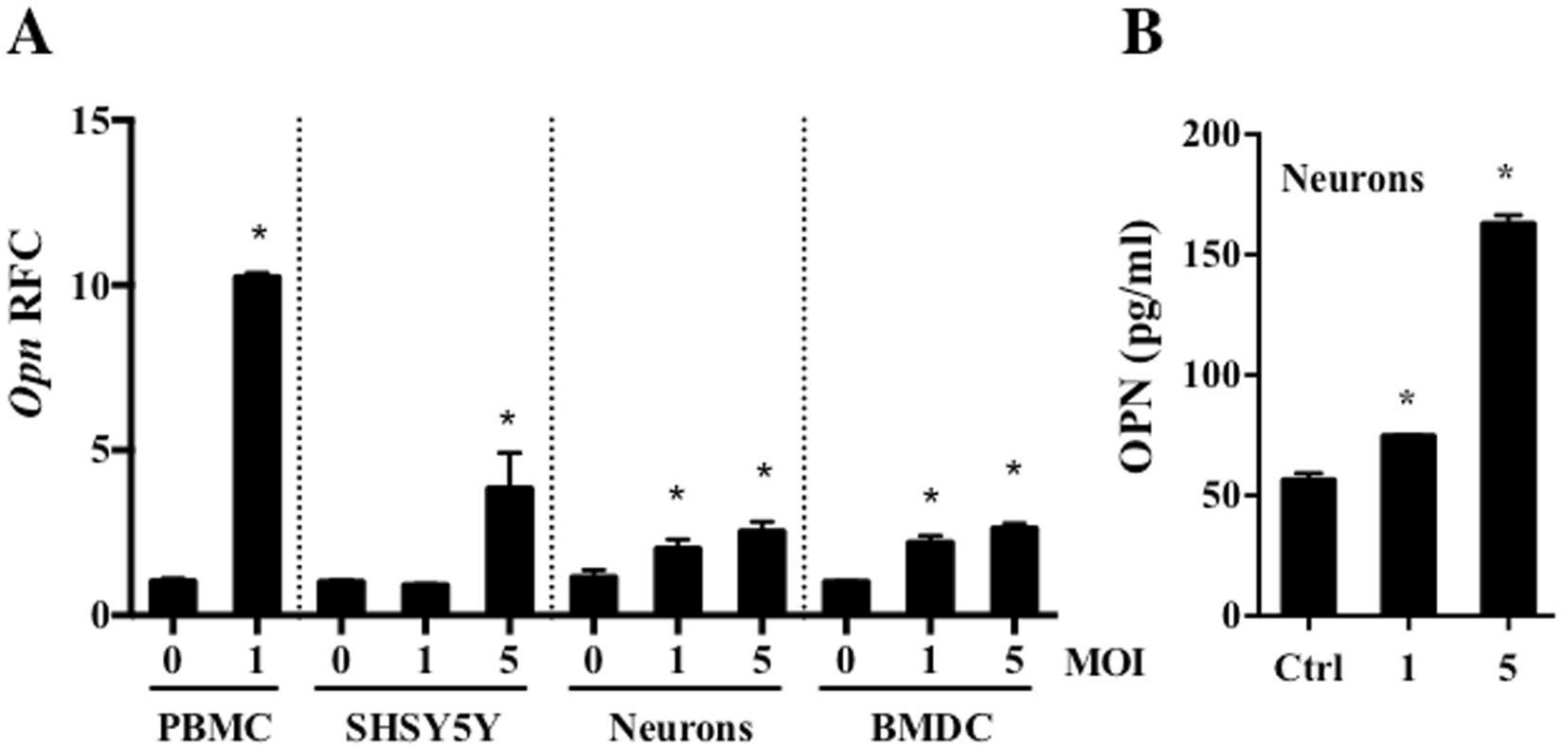
Osteopontin (OPN) is induced following WNV infection in human and mouse cells. (**A**) PBMCs isolated from healthy human volunteers (1 × 10^5^ cells/ml, n = 4) were infected with WNV (MOI = 1) for 24 hr, human neuroblastoma cells (1 × 10^6^ cells/ml, SHSY5Y, n = 3), primary mouse neurons (1 × 10^4^ cells/ml, neurons, n = 3) and mouse bone marrow derived dendritic cells (3 × 10^5^ cells/ml, BMDCs, n = 3) were infected with WNV at MOI of 1 or 5 for 24 hr, and the relative fold change **(**RFC) of *Opn* expression was measured by qPCR, and (**B**) OPN production in the cell medium of primary mouse neurons was measured by ELISA (n = 3). All experiments were performed twice and analyzed using a two-tailed, Student’s *t*-test (*denotes *p* < 0.05).

OPN is induced in various human neuroinflammatory diseases^3, 11–14, 17^. Here, we found that OPN was also induced in human peripheral blood mononuclear cells (PBMC) isolated from healthy human volunteers without a history of WNV infection, following WNV infection *in vitro* (Fig. 1A). In addition, *Opn* expression was increased in WNV-infected mouse bone marrow derived dendritic cells (BMDCs, Fig. 1A). Since WNV is a neurotropic virus^23^ and sOPN is involved in the recruitment of immune cells^3^, we determined if WNV-infected neurons express OPN. For this, human neuroblastoma cells (SH-SY5Y) and murine primary neurons were infected with WNV and *Opn* gene expression was measured by qPCR, and sOPN production in the medium of primary neurons was measured by ELISA. The results showed that the expression of *Opn* was induced in both human and murine neuronal cells (Fig. 1A and B). In summary, these results demonstrated that WNV infection induced OPN expression in both human and mouse cells.

### OPN facilitates WNV infection in mice

**Figure 2:**
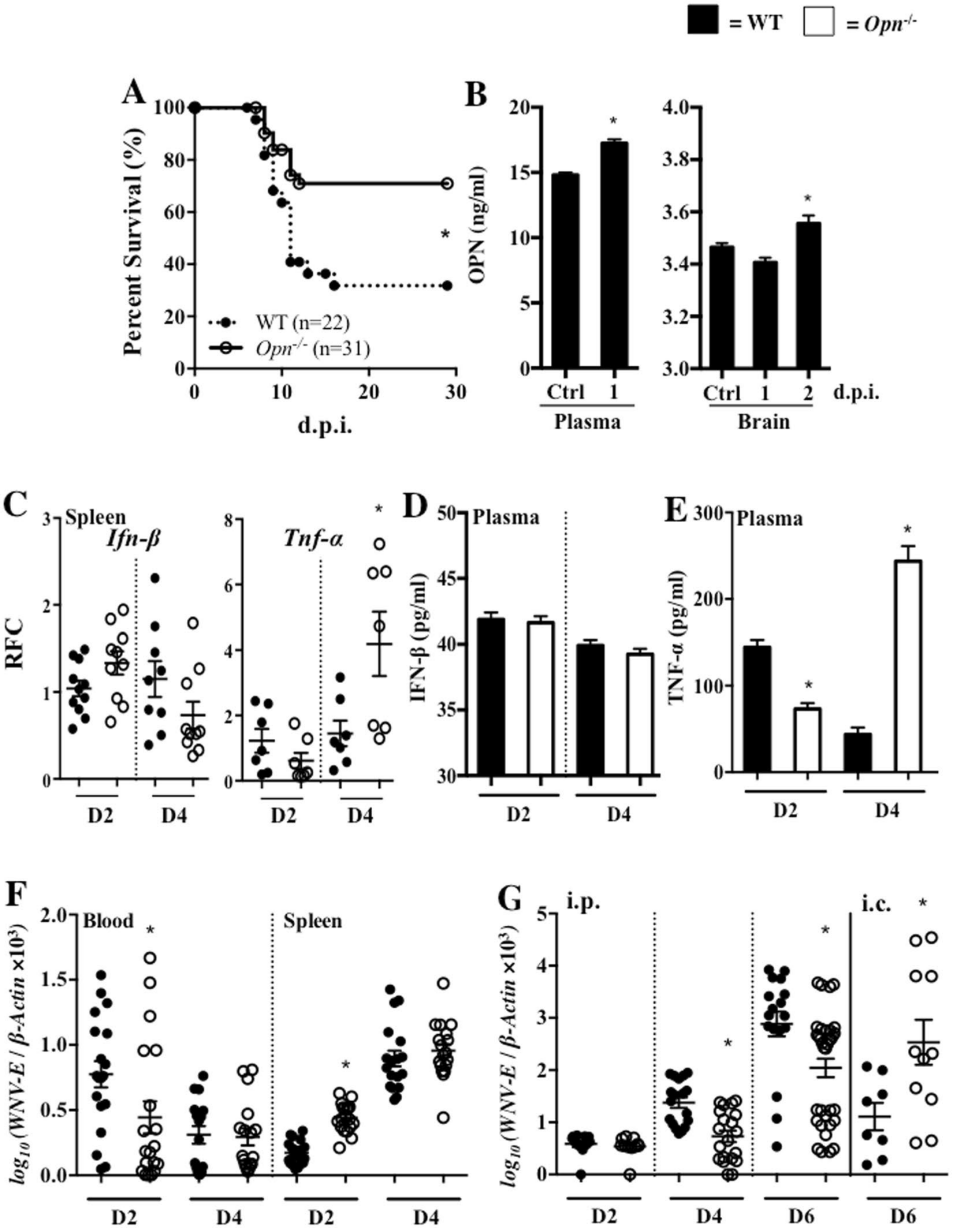
*Opn* ^−/−^ mice infected with WNV have increased survival and reduced viral burden in brain. (**A**) WT and *Opn* ^−/−^ mice were infected with 2,000 PFU of WNV (i.p.) and survival was monitored for 30 days. (**B**) Plasma (left) was collected from WT mice (n = 3) before infection (Ctrl) and on 1 day post infection (d.p.i.) and whole brain homogenates (right) were prepared on 1 and 2 d.p.i. to measure OPN by an ELISA. (**C**) RFC expression of *Ifn*-*β* and *Tnf*-*α* in spleens were measured by qPCR (n = 7–11) normalized to *β*-*actin*. (**D** and **E**) Plasma collected from WT and *Opn* ^−/−^ mice on days 2 (D2) and 4 (D4) p.i. to measure the levels of IFN-β and TNF-α by an ELISA (n = 3). *WNV*-*E* were quantified in blood (n = 16–21) and spleens (n = 18–22) (**F**), and (**G**) brains (n = 10–36) at selected time points (p.i.), and in WT (n = 8) and *Opn* ^−/−^ mice (n = 11) brains that were intracerebrally (i.c.) infected with 20 PFU of WNV on day 6 (D6) p.i. The survival data were analyzed using a Kaplan-Meier log-rank test. All remaining experiments were performed twice and analyzed using a two-tailed, Student’s *t*-test (*denotes *p* < 0.05).

To determine the putative role of OPN in the pathogenesis of WNV *in vivo*, we intraperitoneally (i.p.) infected wild type (WT) and OPN deficient (*Opn* ^−/−^) mice with WNV (2,000 PFU). The survival results showed that approximately 70% of *Opn* ^−/−^ mice and 30% of WT control mice survived WNV infection, suggesting a detrimental role for OPN during WNV infection (Fig. 2A). To further relate the role of OPN to viral burden, we measured OPN levels and viral burden in mice blood, plasma, spleens and brain tissues at various time points post-infection (p.i.). Consistent with the *in vitro* results in Fig. 1A and B, OPN levels were increased in WT mouse plasma at day 1 p.i., and in whole brain homogenates at day 2 p.i. (Fig. 2B). Since OPN has been suggested to regulate type I interferon production^7, 8^ and TNF-α has been implicated in blood brain barrier (BBB) compromise^24^, we measured the expression of *Ifn*-*β* and *Tnf*-*α* in spleens by qPCR. The results showed *Ifn*-*β* mRNA level was not significantly altered between WT and *Opn* ^−/−^ mice at D2 and D4 p.i., but *Tnf*-*α* mRNAs were marginally increased at D4 p.i. (Fig. 2C). Since these molecules circulate in blood and can interact at the BBB interface, we also measured the levels of IFN-β and TNF-α in plasma isolated from WNV-infected WT and *Opn* ^−/−^ mice. Although no significant difference was observed in the levels of IFN-β, interestingly TNF-α displayed a biphasic response in expression, whereby TNF-α levels were reduced at day 2 p.i. and increased at day 4 p.i. (Fig. 2D and E). *WNV*-*envelope (WNV*-*E)* RNA in blood and spleens were comparable between WT and *Opn* ^−/−^ mice at D4 p.i. (Fig. 2F). In contrast, viral RNA in the brain tissues of *Opn* ^−/−^ mice was significantly lower than WT mice at D4 and D6 p.i., while *WNV*-*E* RNA was marginally detected in brain tissues at D2 p.i. with no significant difference between the two groups (Fig. 2G). Viral RNA burden in the brain agreed with the survival results (Fig. 2A), suggesting that OPN may contribute to WNV disease by enhancing infection in the brain. Thus, resistance of *Opn* ^−/−^ mice to WNV infection could be due to two possible mechanisms: either *Opn* ^−/−^ mice brain tissue may be resistant to WNV infection, or fewer viruses invade *Opn* ^−/−^ mice brain parenchyma. To determine if brain-intrinsic antiviral factors were involved in *Opn* ^−/−^ mice, we injected 20 PFU of WNV into the brains of *Opn* ^−/−^ and WT control mice via an intracerebral (i.c) inoculation route, and measured viral RNA in brain tissue on D6 p.i. (Fig. 2G). The results showed there was slightly more viral RNA in the brains of *Opn* ^−/−^ mice than WT controls, excluding the possibility that brain tissue of *Opn* ^−/−^ mice might be intrinsically resistant to WNV replication. In contrast, these results suggest that OPN may facilitate WNV brain invasion.

### *Opn*^−/−^ mice have a tighter BBB following WNV infection

**Figure 3:**
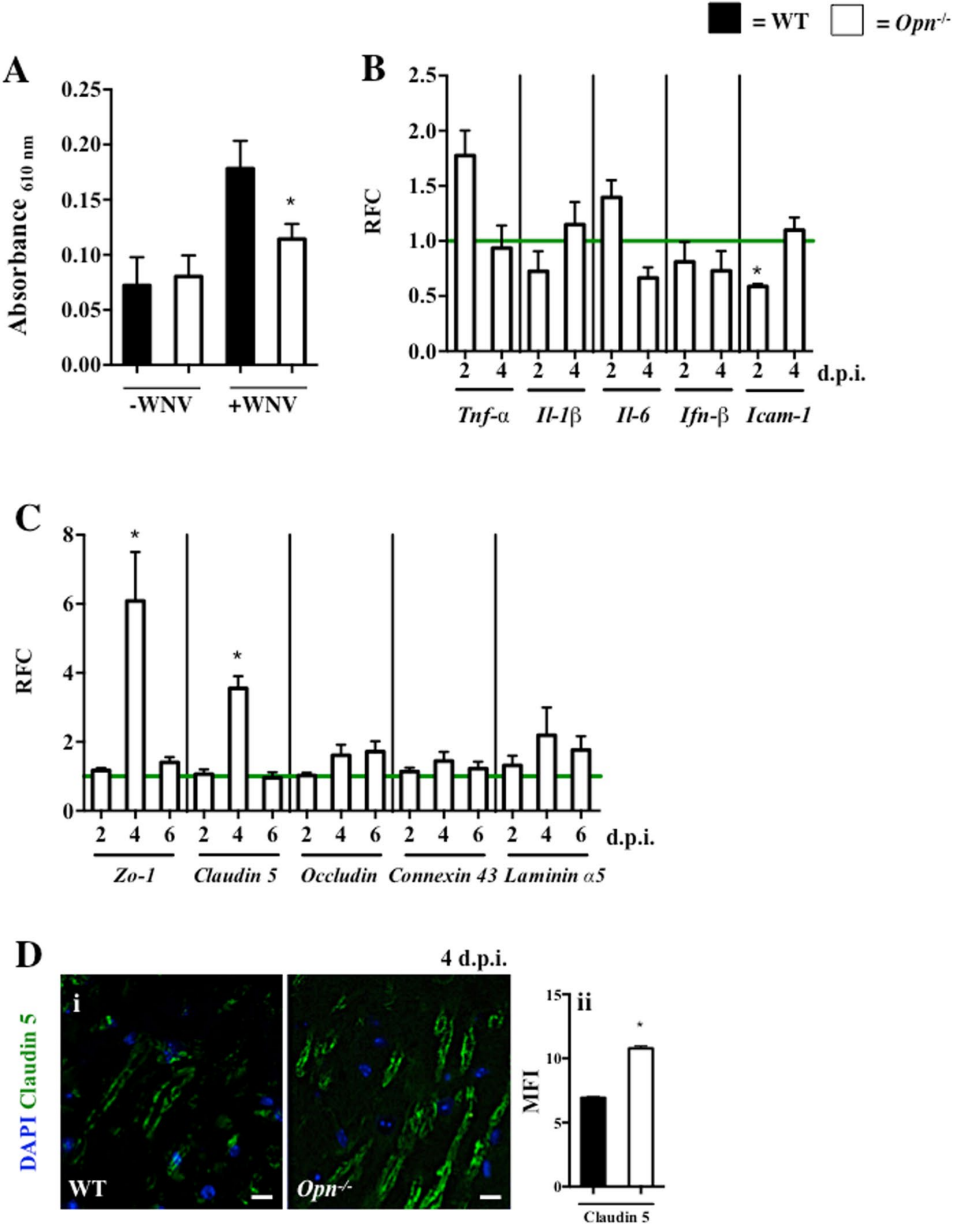
*Opn* ^−/−^ mice have tighter blood brain barriers following WNV infection. WT (n = 21) and *Opn* ^−/−^ (n = 27) mice were infected with WNV (2,000 PFU i.p.) and uninfected WT (n = 12) and *Opn* ^−/−^ (n = 15) mice (PBS i.p., 100 μl) mice were used as absorbance controls. (**A**) Brain infiltrated Evans blue dye was measured with a spectrophotometer on day 4 p.i. (**B**) RFC of *Tnf*-*α*, *Il*-*1β*, *Il*-*6*, *Ifn*-*β* and *Icam*-*1* normalized to *β*-*Actin* in brains of *Opn* ^−/−^ mice compared to WT controls (horizontal green line) on 2 and 4 d.p.i ﻿(n = 4–12)﻿. (**C**) RFC of *Opn* ^−/−^ whole brains were analyzed for the expression of tight junction genes *Zo*-*1*, *Claudin 5*, *Occludin*, *Connexin 43* and *Laminin α5* normalized to *β*-*actin* and compared to WT controls (horizontal green line) on 2, 4, and 6 d.p.i (n = 4–12). (**D**) (i) Immunofluorescence of Claudin 5 (green) and DAPI (blue) in cortical brain sections of WT and *Opn* ^−/−^ mice at 4 d.p.i. following WNV (2,000 PFU) infection, and (ii) mean fluorescent intensity (MFI) of Claudin 5. All experiments were performed twice and analyzed using a two-tailed, Student’s *t*-test (*denotes *p* < 0.05). Scale bar = 10 μm.

The BBB is an important physiological barrier for protecting the brain against various insults, including WNV and other neuroinvasive pathogens^23, 25^. We next sought to determine if the BBB is tighter in *Opn* ^−/−^ mice than WT mice without or with WNV infection with an Evans blue assay. Similar levels of Evans blue dye was observed in brain tissues from uninfected *Opn* ^−/−^ and WT controls, which suggests that OPN does not contribute to BBB compromise. However, following WNV infection, *Opn* ^−/−^ mice had reduced Evans blue dye brain tissue integration as compared to WT controls (Fig. 3A), suggesting *Opn* ^−/−^ mice have a tighter BBB following infection. To determine if there were any changes in inflammatory or antiviral responses between WT and *Opn* ^−/−^ brain tissues, we used qPCR to measure the expression of *Tnf*-*α*, *Il*-*1β*, *Il*-*6* and *Ifn*-*β* with the results indicating no significant difference in the expression of these genes (Fig. 3B). Interestingly, the gene expression of an endothelial cell marker involved in leukocyte extravasation, intracellular adhesion molecule-1 (*Icam*-*1*), was significantly reduced in the brain tissues of *Opn* ^−/−^ mice at 2 d.p.i., suggesting defective leukocyte extravasation into the brain of *Opn* ^−/−^ mice at the early stage of infection (Fig. 3B). In addition, we also evaluated mRNA expression of BBB tight junction related genes *Zo*-*1*, *Claudin 5*, *Occludin*, *Connexin 43* and *Laminin α5* and found *Zo*-*1* and *Claudin 5* were significantly higher in brain tissues of *Opn* ^−/−^ mice on D4 p.i., with a trend in higher expression of *Occludin*, *Connexin 43* and *Laminin α5* (Fig. 3C). We also confirmed the expression of Claudin 5 at the protein level by immunofluorescence of whole brain tissue sections, which showed increased mean fluorescent intensity (MFI) of Claudin 5 localized to blood vessels that innervated cortical brain parenchyma in *Opn* ^−/−^ mice compared to WT controls (Fig. 3D). Collectively, these results indicated that *Opn* ^−/−^ mice have a tighter BBB than WT control mice following WNV infection.

### *Opn*^−/−^ mice have less PMN-brain infiltration following WNV infection

**Figure 4:**
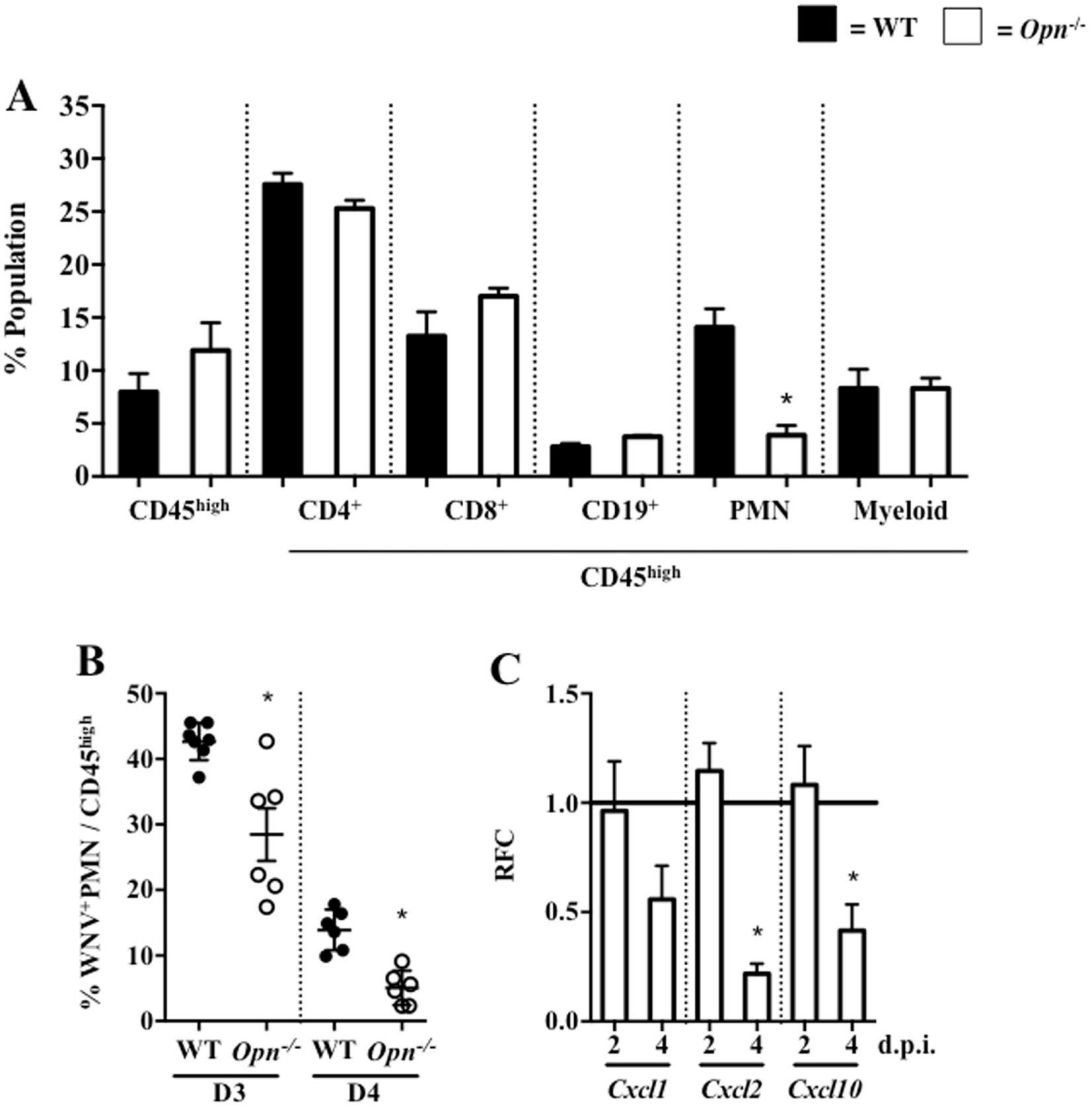
*Opn* ^−/−^ mice have reduced PMN-brain infiltration following WNV infection. WT and *Opn* ^−/−^ mice were infected with 2,000 PFU (i.p.). (**A**) Brain infiltrating leukocytes were quantified by flow cytometry on day 4 p.i., including total infiltrating leukocytes (CD45^high^), CD4^+^ cells (CD4^+^/CD45^high^), CD8^+^ cells (CD8^+^/CD45^high^), CD19^+^ cells (CD19^+^/CD45^high^), PMNs (Ly6G^+^CD11b^+^/CD45^high^) and myeloid cells (Ly6G^-^CD11b^+^/CD45^high^); n = 5–7 per group. (**B**) Brain-infiltrating WNV^+^ PMN (% WNV^+^Ly6G^+^/CD45^high^) were quantified on days 3 (D3) and 4 (D4) p.i. (n = 5–7). Brains were collected (2 and 4 d.p.i.), subjected to qPCR and the RFC of *Opn* ^−/−^ compared to WT controls (horizontal black line, n = 6–12) for *Cxcl1*, *Cxcl2* and *Cxcl10* were normalized to *β*-*actin*. Experiments were performed at least twice and analyzed using a two-tailed, Student’s *t*-test (*denotes *p* < 0.05).

To further investigate the contributing role of OPN during WNV-mediated BBB permeability, we examined immune cell infiltration and WNV-E antigens in the brains of *Opn* ^−/−^ and WT control mice by flow cytometry. The results showed that among all the measured brain-infiltrating leukocytes, PMNs were the predominant cell type that were reduced (approximately 70%) in *Opn* ^−/−^ mice compared to WT mice (Fig. 4A and Supplemental Fig. 1). To further determine if OPN recruits PMNs carrying WNV into the brain, we infected WT and *Opn* ^−/−^ mice with WNV (2,000 PFU, i.p.) and isolated their brains on D3 and D4 p.i. for flow cytometric analysis. We found that the percentages of WNV-infected PMNs were reduced in the brains of *Opn* ^−/−^ mice during the early infection course compared to WT controls (Fig. 4B). Since chemokines are responsible for PMN recruitment^26^, we measured expression of chemokines in brain tissues of WT and *Opn* ^−/−^ mice. We found that PMN chemoattractant genes *Cxcl2* and another chemokine *Cxcl10*, had significantly reduced expression in *Opn* ^−/−^ mice compared to WT controls at 4 d.p.i. (Fig. 4C), which may be attributed to milder viral infection in the brain. In summary, these results suggest that PMN infiltration is reduced in the brains of *Opn* ^−/−^ mice, which may be in part due to reduced PMN chemoattractant gene expression.

### sOPN recruits WNV-infected PMNs into the brain enhancing disease severity

**Figure 5:**
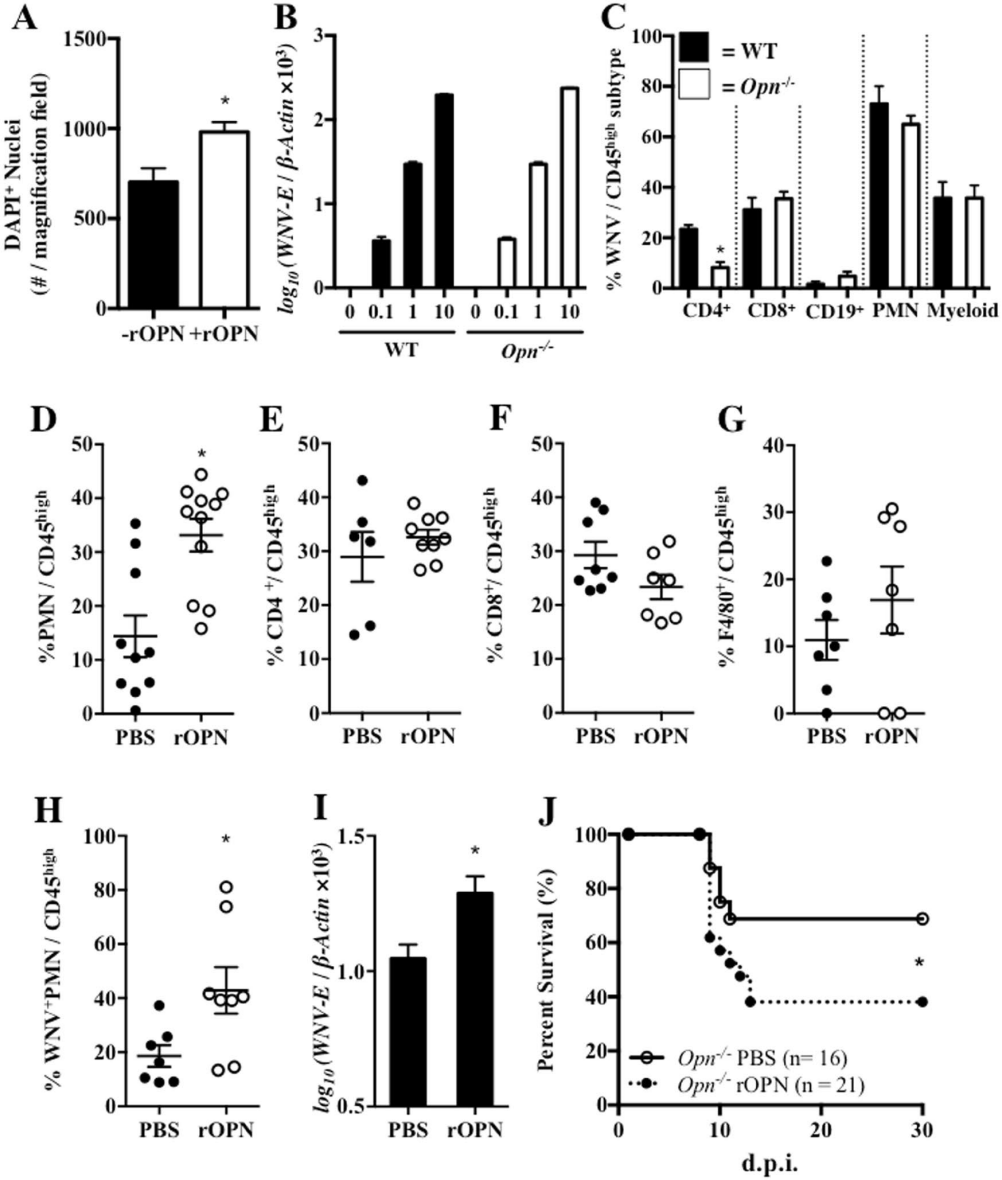
Recombinant OPN recruits PMNs *in vitro* and *in vivo* and increases WNV infection in *Opn* ^−/−^ mice. (**A**) Transwell migration assay using bone marrow derived PMNs isolated from WT mice (n = 3) and recombinant mouse OPN (rOPN, 2 μg/ml). (**B**) Bone-marrow derived PMNs isolated from WT and *Opn* ^−/−^ mice (n = 4) were infected *in vitro* with WNV for 24 hr, followed by qPCR analysis to measure *WNV*-*E* relative to *β*-*Actin*. (**C**) WT and *Opn* ^−/−^ mice were infected with WNV (2,000 PFU) and the percentage (%) of WNV^+^ expressing brain infiltrating leukocytes were profiled for total populations of CD4^+^ cells (CD4^+^/CD45^high^), CD8^+^ cells (CD8^+^/CD45^high^), CD19^+^ cells (CD19^+^/CD45^high^), PMNs (CD11b^+^Ly6G^+^/CD45^high^) and myeloid cells (CD11b^+^Ly6G^−^/CD45^high^); n = 5–7 per group. *Opn* ^−/−^ mice were pretreated with rOPN (50 ng, i.c.) or a PBS-vehicle control and 1 hr later were infected with WNV (2,000 PFU, i.p.). Brain-infiltrating leukocytes (n = 6–11) were quantified by flow cytometry on day 4 p.i.; (**D**) PMNs (Ly6G^+^CD11b^+^/CD45^high^), (**E**) CD4^+^ cells (CD4^+^/CD45^high^), (**F**) CD8^+^ cells (CD8^+^/CD45^high^), and (**G**) macrophages (F4/80^+^/CD45^high^). Brains were collected on day 4 p.i. for flow cytometric analysis and qPCR measurement of WNV. (**H**) Percent (%) population of WNV^+^PMNs (WNV^+^Ly6G^+^) in total infiltrating leukocytes (CD45^high^) by flow cytometric analysis (n = 7–8). (**I**) *WNV*-*E* relative to *β*-*Actin* was analyzed by qPCR in brains of *Opn* ^−/−^ mice with PBS (n = 6) or rOPN (n = 7) i.c. supplement. (**J**) Survival of *Opn* ^−/−^ mice either pretreated i.c. with rOPN (n = 21) or PBS (n = 16) for 1 hr, followed by infection with 2,000 PFU of WNV (i.p.). The survival data were analyzed using a Kaplan-Meier log-rank test. All remaining experiments were performed twice and analyzed using a two-tailed, Student’s *t*-test (*denotes *p* < 0.05).

OPN has been shown to recruit PMNs *in vitro* ^9^. In our study, we confirmed that recombinant OPN (rOPN) could attract PMNs in a transwell migration assay (Fig. 5A), suggesting that along with reduced expression of *Cxcl2*, reduced PMN infiltration in brains of *Opn* ^−/−^ mice may also be due to lack of sOPN. In addition, we confirmed that deficiency of OPN did not alter PMN permissiveness to WNV infection by qPCR (Fig. 5B). We have previously demonstrated that PMNs were very permissive to WNV infection^20, 27^. In agreement with these reports, here we also identified PMNs as the primary carriers of WNV during neuroinvasive infection in mice, as approximately 75% of total infiltrated PMNs were WNV^+^ within both WT and *Opn* ^−/−^ mice (Fig. 5C). Therefore, it is possible that sOPN might recruit WNV-infected PMNs into brain, enhancing WNV neuroinvasion. To test this hypothesis *in vivo*, we injected rOPN (50 ng, i.c.) or an equal volume of PBS into the brains of *Opn* ^−/−^ mice and 1 hr later infected them with WNV (2,000 PFU, i.p.). Mice were euthanized and brains were isolated on D4 p.i., a time point when the BBB was largely compromised as indicated by the Evans blue assay (Fig. 3A) and total brain infiltrated leukocytes were quantified by flow cytometry. We found that infiltrated PMNs were considerably increased following rOPN supplement in *Opn* ^−/−^ mice brains, while the infiltration of CD4^+^ cells, CD8^+^ cells, and macrophages were not significantly altered (Fig. 5D–G), suggesting that rOPN can selectively recruit PMNs into brain following WNV infection. In complement with this, rOPN-supplemented *Opn* ^−/−^ mice had increased percentage of WNV-infected PMN infiltration into their brains (Fig. 5H) and WNV burden was significantly higher in brains of rOPN-supplemented *Opn* ^−/−^ mice (Fig. 5I), which resulted in dramatically reduced survival of rOPN-supplemented *Opn* ^−/−^ mice compared to PBS-supplemented *Opn* ^−/−^ mice (Fig. 5J). Collectively, our results suggested that sOPN could recruit WNV-infected PMNs into the brain, facilitating WNV neuroinvasive disease.

## Discussion

Understanding the mechanisms by which WNV invades the CNS is a prerequisite for developing specific antiviral therapeutics to combat WNV neuroinvasive disease and associated long-term sequelae^1, 2^. In theory, WNV can enter the brain through multiple routes, including diffusion through endothelial tight junctions, direct infection of endothelial cells, infected leukocytes that ‘Trojan horse’ WNV into the CNS, infection of olfactory neurons, and/or direct axonal retrograde transport from infected peripheral neurons^23, 28–31^. Infected T cells and macrophages have been suggested as ‘Trojan horse’ WNV carriers into the CNS^29, 32, 33^, while our previous report demonstrated that WNV replicates more efficiently in human and mouse PMNs^20^. In addition, both clinical and experimental mouse data show that PMNs are the predominant infiltrating leukocyte subpopulation in the CNS during the early-stage of WNV infection^22, 34, 35^. Therefore, it is possible that a small number of viral particles enter the brain and infect neuronal cells and immune cells to produce chemoattractant molecules that could trigger an influx of WNV-infected PMNs into the brain, paradoxically making infection more severe. Understanding the host factors necessary for the regulation of PMN recruitment into the CNS and whether infiltrating PMNs carry WNV into the CNS becomes essential to elucidate the contribution of PMNs in WNV neuroinvasion.

In this study, we found that the expression of OPN was induced following WNV infection in human and mouse neuronal cells *in vitro*. In line with this, neurons have been described to be major producers of OPN in HIV-associated neurocognitive disorders^36^, while other CNS-resident cells including astrocytes^37^ and microglia^38^ induce OPN expression following glioma-associated activation, suggesting OPN may be involved in the pathogenesis of other neurodiseases. Here, we revealed a novel role for OPN in enhancing WNV neuroinvasion in mice, as shown by reduced viral burden in the brain and increased survival of WNV-infected *Opn* ^−/−^ mice compared to WT controls. Consistent with the previously reported role of sOPN in PMN recruitment^9^, we found that PMN infiltration was significantly reduced in the brains of *Opn* ^−/−^ mice compared to WT mice. By using a transwell migration assay and supplementing rOPN into the brains of *Opn* ^−/−^ mice, we further confirmed that OPN could recruit WNV-infected PMNs into the brain, resulting in a higher viral burden and reduced survival. Therefore, we concluded that OPN facilitates WNV neuroinvasive infection through the recruitment of WNV-infected PMNs.

Reduced leukocyte infiltration in particular with WNV-infected PMNs in the brains of *Opn* ^−/−^ mice following WNV infection, was negatively correlated with viral burden in brain tissue during an early stage of infection (D4 p.i.). Although antiviral functions of PMNs, such as NETs and reactive oxygen species (ROS) production were not studied in this report, PMNs isolated from WT and *Opn* ^−/−^ mice were both susceptible to equal viral loads and had similar capacity to transport WNV into the brain, which rules out defective immunity in *Opn* ^−/−^ PMNs. Our results confirmed that WNV induced OPN expression in neuronal cells, which indicates sOPN produced by WNV-infected neurons could play an important role in recruiting WNV-infected PMNs into the brain tissue following initial WNV brain entry. In support of this, i.c. rOPN supplement significantly increased WNV-infected PMN infiltration into the brains of *Opn* ^−/−^ mice compared to i.c. PBS supplemented controls. In addition, the expression of the PMN-attracting chemokine *Cxcl2* was also diminished in the brains of WNV-infected *Opn* ^−/−^ mice, suggesting OPN may not be the sole factor responsible for reduced PMN infiltration in *Opn* ^−/−^ mice following WNV infection.

Although PMNs are necessary to combat WNV infection during a later time point of neuroinvasive WNV infection^20^, viral load was substantially reduced in *Opn* ^−/−^ mice early during infection (D4), which may be due to reduced PMN brain influx, contributing to enhanced survival. Indeed, the BBB was tighter in *Opn* ^−/−^ than WT mice following WNV infection, possibly due to the reduced influx of leukocytes and neuroinflammation, as robust immune cell infiltration could also interrupt the integrity of the BBB^25, 39, 40^. Furthermore, OPN did not appear to contribute to BBB compromise directly in our mouse model, while *Opn* ^−/−^ mice had reduced gene expression of *Icam*-*1* and TNF-α levels early in the course of infection (D2 p.i.). ICAM-1 has been shown to regulate the integrity of the BBB in the mouse model of WNV infection^41^, which suggests other pathways that affect the integrity of the BBB may be also inhibited in *Opn* ^−/−^ mice directly or indirectly following infection. In addition, other than contributing to BBB compromise^24^, TNF-α has also been shown to be involved in the promotion of WNV clearance^42^ and to protect against WNV-induced neuronal apoptosis^43^, indicating multiple functions of TNF-α in the immune response that can support or inhibit viral-induced neurodisease, which requires further studies.

In conclusion, our results show that reduced PMN infiltration into the brain partially limits WNV neuroinvasion, suggesting OPN might serve as a potential therapeutic target to reduce WNV neuroinvasion.

## Methods

### Ethics statement and biosafety

Written informed consent was obtained from all human volunteers prior to their inclusion in this study. The protocol for human subject has been approved by the University of Southern Mississippi (USM) Institutional Review Board. All animal experimental procedures were approved by the Institutional Animal Care and Use Committee at USM. All *in vitro* experiments and animal studies involving live WNV were performed by certified personnel in biosafety level 3 (BSL3) laboratories following standard biosafety protocols approved by the USM Institutional Biosafety Committee.

### Viruses and cell culture

WNV isolate (CT2741), kindly provided by John F. Anderson, was propagated one time in Vero cells (ATCC CCL-81) and titered by a Vero cell plaque assay^44^. Vero (ATCC CCL-131) were maintained in Dulbecco’s modified Eagle medium (DMEM) containing 1% L-glutamine (L-glu), 1% penicillin-streptomycin (Pen/Strep), and 10% FBS. SHSY5Y cells (ATCC CRL-2266) were maintained in Eagle’s minimal essential medium (EMEM) and F12 medium (1:1) containing 10% FBS. Mouse BMDCs were cultured in DMEM containing 10% FBS, 2% plasmacytoma cell medium containing GM-CSF (J588L), 1% Pen/Strep, 1% L-glu and 50 μM of β-mercaptoethanol. Mouse primary neurons were cultured in DMEM:F12 (1:1) medium containing 1% Pen/Strep, 10% FBS, 1% L-glu, and glucose (4.5 g/l) and Neurobasal^®^-A media (Life Technologies) containing 2% B-27, 10% FBS, 1% L-glu and 1% Pen/Strep. Polymorphonuclear neutrophils (PMNs) were cultured in RPMI-1640.

Human peripheral blood mononuclear cells were isolated using Ficoll-Paque PLUS (GE Healthcare Life Sciences)^20^. Murine BMDCs were isolated and cultured from femurs of WT or *Opn* ^−/−^ mice (3 to 6 month-old)^45^, primary neuronal cells were isolated from adult (6–12 month-old) mice, triturated in Papain (2 mg/ml) and cultured on poly-ornithine pre-treated plates^46, 47^, and PMNs were isolated and cultured^48^, following previously published procedures. Purity of isolated PMNs was measured by flow cytometry as gated CD45^+^CD11b^+^Ly6G^+^ populations.

### Animal Studies

C57BL/6J wild-type (WT) and *Opn* ^−/−^ (C57BL/6J background) mice breeding pairs were purchased from The Jackson laboratory (Bar Harbor, ME). Mice were bred in a USM animal facility clean room. Seven to nine week-old, sex-matched WT and *Opn* ^−/−^ mice were inoculated intraperitoneally (i.p.) with 2,000 plaque-forming units (PFUs) of WNV in 100 µl of PBS containing 1% gelatin^20^. Infected mice were monitored daily for morbidity and mortality for 30 days. Blood samples were collected by retro-orbital bleeding on D1–4 p.i. for viremia and *Opn* measurement by qPCR and mice were randomly euthanized on D2, D4, and D6 p.i. to collect spleen and brain tissues for gene expression and flow cytometric analysis. For intracerebral (i.c.) injections of WNV (20 PFU/mouse in 20 µl 1% gelatin) and recombinant OPN (rOPN; 50 ng/mouse in 20 µl in PBS) were performed on 8-week old, sex-matched mice anesthetized with 30% isoflurane in isopropanol^27^. For rOPN studies, one-hour post rOPN supplement, mice were inoculated with 2,000 PFU of WNV per mouse (i.p.) and monitored for 30 days.

### Quantitative PCR (qPCR) and Enzyme-linked Immunosorbant Assays

Mouse tissues and cells were collected for total RNA extraction with TRIreagent (Molecular Research Center, Inc.) and converted into the first strand cDNA using the iSCRIPT^TM^ cDNA synthesis kit (Bio-Rad). qPCR assays were performed using iTAQ^TM^ polymerase supermix for probe-based assays (Bio-Rad) or iQ™ SYBR® Green Supermix polymerase (Bio-Rad). WNV-envelope (*WNV*-*E*) gene and mouse cellular gene primers and probes sequences were adapted according to previous publications: *Opn* ^49, 50^, *WNV*-*E* ^51^, *β*-*Actin* ^20, 52^, *Zo*-*1* ^53^, *Claudin 5* ^54^, *Occludin* ^55^, *Connexin 43* ^56^, *Ifnβ* ^57^, *Tnf*-*a* ^58^, *Il*-*1β* ^59^, *Il*-*6* ^60^ and *Icam*-*1* ^60^,*Cxcl1* ^61^, *Cxcl2* ^62^, and *Cxcl10* ^63^. Primers were designed for murine *Laminin α5*, forward 5′-TGGCTCACAGATGGCTCCTA-3′, and reverse 5′-CACTTGGATCGCCTTGCTGG-3′. Analyses were performed using either the 2^−ΔΔCT^ method, normalized to *β*-*Actin* and represented as relative fold change (RFC) or the ratio of the absolute gene copy number of *WNV*-*E* to *β*-*Actin*. All primers and the *WNV*-*E* probe were synthesized by Integrated DNA Technologies or Applied Biosystems. Levels of OPN were quantified in murine plasma samples and culture media by using a mouse OPN ELISA kit (ENZO Life Sciences) following manufactures recommendations.

### Evans Blue Assay

WT and *Opn* ^−/−^ mice were ﻿﻿mock-infected with PBS or infected with 2,000 PFU of WNV (i.p.), and 800 µl of 2% Evans Blue dye (EBD) in PBS was injected (i.p.) on day 4 p.i. One hour after EBD injection, mice were transcardially perfused with ice-cold PBS. EBD in whole brain tissue was quantified using a spectrophotometer after DMSO homogenization (Bio-Rad Smart Spec 3000^TM^). All samples were normalized to control brains (with EBD, without WNV infection) for each genotype group (WT or *Opn* ^−/−^).

### Migration Assays

PMN migration assays were performed as previously described^9^, with additional modifications. Briefly, WT and *Opn* ^−/−^ PMNs in RPMI-1640 were added to the inserts of transwells (3 μm pore, Corning), while RPMI-1640 with or without rOPN (2 μg/ml, R&D Systems) was added to the bottom of each well. The cells were incubated for 8 hr in a 37 °C, 5% CO_2_ incubator. Migrated PMNs were quantified by counting the number of DAPI positive cells/magnification field that were 4% PFA fixed in the transwell interface using a confocal LSM 510 microscope (Zeiss) at 10× magnification.

### Immunofluorescence assay

Mouse brains isolated after ice-cold PBS perfusion were 4% PFA fixed overnight, dehydrated, and paraffin-embedded for sectioning. Midsagittal sections (10 µm) were cut (American Optical Spencer 820 Microtome) and mounted. For immunostaining, sections were dried for 2 hr at 45 °C and rehydrated for 5 minutes in 100%, 95%, and 70% ethanol solutions followed by antigen retrieval by boiling in 5% sodium citrate for 10 minutes (50% power) in a microwave. Sections were probed with anti-Claudin 5 conjugated to flourophore 488 antibody (Thermo Fisher), mounted with Vectashield® mounting medium for fluorescence with DAPI, and imaged under a confocal LSM 510 microscope (Zeiss) at 63× magnification. Mean fluorescent intensity was quantified using ImageJ software^64^.

### Flow cytometry

Procedures for brain cell preparation followed our previous report^51^. Cells (1 × 10^6^ cells/ml) were probed with antibodies against CD45, Ly6G, CD11b, CD4, CD19, CD8 and F4/80 and analyzed with a flow cytometer (LSRFortessa, BD Bioscience) using version 6.0 FACSDiva™ software (BD Biosciences). All cell surface antibodies were purchased from eBioscience. Anti-WNV-Envelope (E) primary antibody (Abcam) and FITC-conjugated anti-mouse IgG secondary antibody (eBioscience) were used for viral detection. Unstained and single color compensation controls were used in parallel.

### Enzyme-linked Immunosorbent Assay

Levels of OPN, IFN-β and TNF-α were measured using ELISA kits purchased from ENZO Life Sciences or Thermo Fisher following manufactures recommendations.

### Statistical analyses

All data were analyzed using a two-tailed Student’s *t*-test and survival curves were analyzed using a Kaplan-Meier analysis. All statistical analyses were done by using GraphPad Prism software (version 6.0), with *p* < 0.05 considered statistically significant.

## Electronic supplementary material

Supplemental materials
