# Supplementary material for: Osteopontin facilitates West Nile virus neuroinvasion via neutrophil “Trojan horse” transport
Source: Sci Rep. 2017 Jul 5;7:4722. doi: 10.1038/s41598-017-04839-7 (PMC5498593; doi:10.1038/s41598-017-04839-7)

Supplemental Materials

Osteopontin facilitates West Nile virus neuroinvasion via neutrophil "Trojan horse" transport

Amber M. Paul, Dhiraj Acharya, Laurel Duty, E. Ashley Thompson, Linda Le, Dobrivoje S. Stokic, A. Arturo Leis and Fengwei Bai

Supplemental Figure 1. Flow cytometric gating scheme. Brain-infiltrating cells were profiled by gating $CD45^{high}$ populations from total events recorded. The subpopulations within $CD45^{high}$ population including $CD4^{+}$ cells ($CD4^{+}$ / $CD45^{high}$), $CD8^{+}$ cells ($CD8^{+}$ / $CD45^{high}$), $CD19^{+}$ cells ($CD19^{+}$ / $CD45^{high}$), PMNs ($CD11b^{+}Ly6G^{+}$ / $CD45^{high}$) and myeloid cells ($CD11b^{+}Ly6G^{-}$ / $CD45^{high}$) were further analyzed. Percentage of WNV antigen positive cells were also gated within each subpopulation (CD4, CD8, CD19, PMNs and myeloid cells) with an example histogram for the percentage of WNV^{+}PMN in total infiltrating PMNs is shown. In addition, the % of $WNV^{+}Ly6G^{+}$ (WNV-PMNs) in total $CD45^{high}$ cells were analyzed as shown.

Supplemental Figure 1.

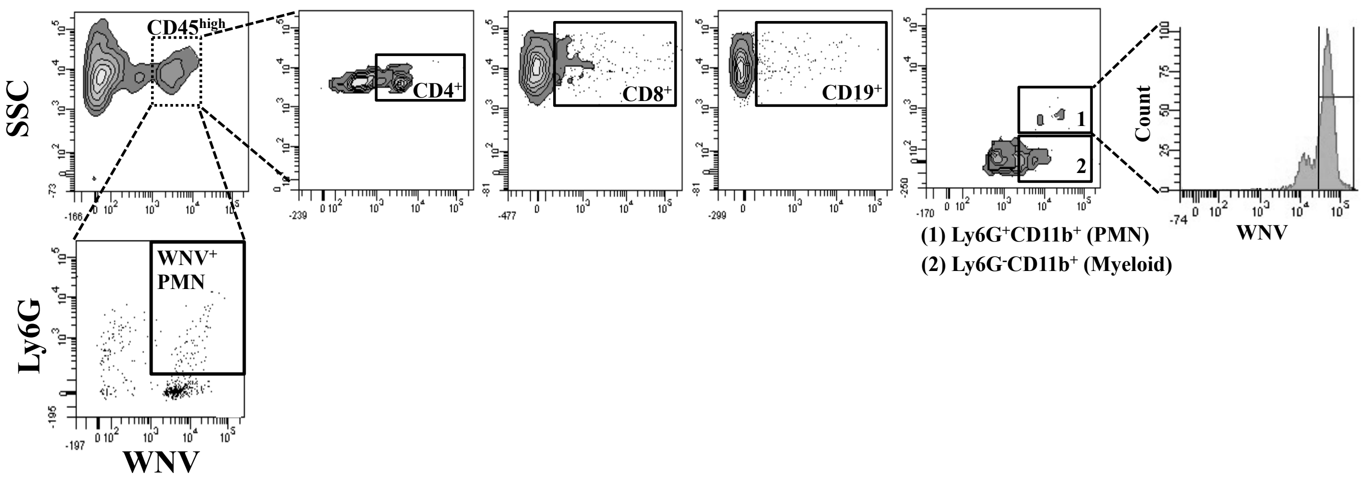

Supplement: Supplementary file 1 — Supplemental materials [file 41598_2017_4839_MOESM1_ESM.pdf]
